# Percutaneous coronary intervention-associated *Actinomyces oris*

**DOI:** 10.1016/j.idcr.2020.e00929

**Published:** 2020-08-08

**Authors:** Walaa Saeed, Mohammad Adam, Tasneem A. Abdallah, Ali S. Omrani

**Affiliations:** a Department of Medicine, Hamad General Hospital, Hamad Medical Corporation, Doha, Qatar; b Division of Infectious Diseases, Department of Medicine, Hamad Medical Corporation, Doha, Qatar; c Communicable Disease Center, Hamad Medical Corporation, Doha, Qatar

**Keywords:** *Actinomyces oris*, Actinomycosis, PCI, Cardiac

## Abstract

Coronary artery interventions are safe procedures yet have a risk of stent infection, bacteremia and sepsis, events that are rare but with high morbidity and mortality sequel. A few prior cases had reported post percutaneous coronary intervention (PCI) infections, abscesses and sepsis due to *Staphylococcus aureus*, followed by *Pseudomonas aeruginosa.* Cardiac Actinomyces infections are extremely rare. Here we report a case of a 50 year old patient who developed a post intervention *Actinomyces oris* epicardial abscess occluding right coronary artery with subsequent bacteremia eventually requiring open heart surgery. He was treated during and thereafter with IV penicillin and ceftriaxone for almost 8 weeks. We highlight during this review the available literature regarding risk factors, the possible theories of acquiring such bacterium at this unusual site as well as our patient’s course and treatment outcome.

## Introduction

*Actinomyces* species are anaerobic, Gram-positive, filamentous bacteria which are normal commensals of the human oral cavity, gut and female genital tract. More than 20 species have been described, with *Actinomyces israelii* being the species mostly commonly associated with human infections [1,2]. *Actinomyces* infections are usually the result of disruption of mucosal barriers secondary to trauma, surgical procedures or foreign bodies, leading to bacterial invasion of deeper tissues, and rarely, the blood stream [3,4]. Actinomycosis is usually categorized according to the body site affected as oro-cervico-facial, thoracic and abdomino-pelvic forms [1,4]. Typical clinical presentation is one of subacute progression with abscess formation and eventual fistulization into an internal anatomic space or external sinus drainage. Clinical and radiological presentation may mimic malignant diseases or chronic infections such as tuberculosis [4,5].

The risk of infective complications in association with percutaneous coronary interventions (PCI) is generally very low [6]. Though rare, PCI-associated infections result in high rates of morbidity and mortality [7]. Clinical presentation is usually in the form of recurrent stent thrombosis or abscess formation, or occasionally as blood stream infection or aneurysm [8]. *Staphylococcus aureus* and *Pseudomonas aeruginosa* are the bacteria most frequently implicated in coronary stent infections [[9], [10], [11], [12]].Such infections typically present within a few days or weeks from the procedure [10]. Late presentations are considered rare [13,14]. We herein report a case of iatrogenic pericardial actinomycosis presenting four months after percutaneous coronary artery stenting.

## Case report

A 50 year old man with history of type 2 diabetes mellitus and past tobacco smoking underwent elective balloon angioplasty of the right coronary artery (RCA) in October 2018. The procedure was unsuccessful due to complete occlusion of the RCA lumen. A follow up elective procedure was performed 3 months later during which successful retrograde canalization of the RCA was achieved and three drug-eluting stents were placed.

**Fig. 1:**
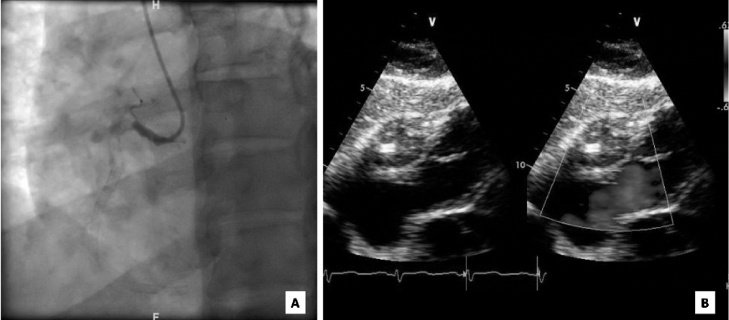
(a) Coronary angiogram showing complete right coronary artery occlusion. (b) Trans-thoracic echocardiogram with a tissue like mass seen in the lateral annulus of the tricuspid valve measuring 41 by 29 mm with an area of calcification in the center.

**Fig. 2:**
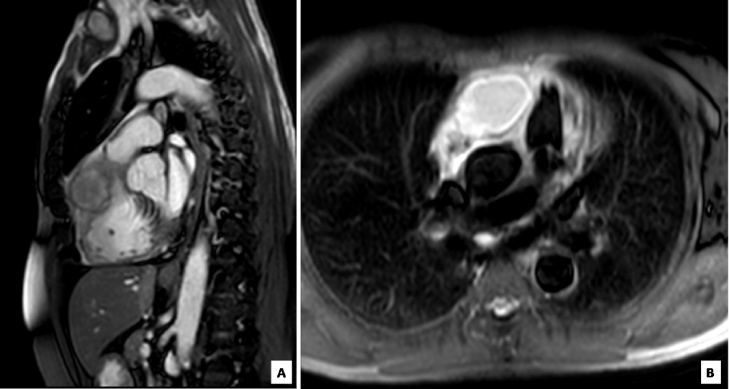
Sagittal (a) and transverse (b) magnetic resonance images of the heart showing a well-defined epicardial cystic lesion with a lobulated outline and thin internal septations.

Three months after the procedure, he presented with a history of left sided pleuritic chest pain radiating to the left arm. The pain was exaggerated by physical exertion and breathing, and was relieved by rest. A working diagnosis of unstable angina was made on the basis of ECG showing left axis deviation and poor R wave progression with stable serial serum troponin levels. Coronary angiography showed complete occlusion of the RCA stent, in addition to progression of coronary artery disease to involve three additional vessels including the left main trunk (Fig. 1A). Transthoracic echocardiography showed evidence of a 41 by 29 mm epicardial mass near the lateral annulus of the tricuspid valve (Fig. 1B). The lesion was not seen on previous echocardiography. Cardiac magnetic resonance imaging (MRI) showed the mass to be of cystic nature encasing the proximal to distal stented segments of the RCA (Fig. 2A and B). The patient subsequently developed recurrent nitrate-responsive chest pain with fever and rigors. His oral temperature was 38.7 degrees Celsius with stable blood pressure and pulse rate. ECG showed new ST segment elevation in the inferior lead with an associated rise in serum troponin T from 91 ng/L to 1140 ng/L. Blood work-up was also significant for C- reactive protein (CRP) 151 mg/L and procalcitonin 16.5 ng/mL.

Empiric therapy with intravenous piperacillin-tazobactam and vancomycin was started. Five days later, a provisional blood culture report described the presence of filamentous Gram-positive bacteria; later identified using automated Matrix Assisted Laser Desorption Ionization Time-of-Flight (MALDI-TOF) mass spectrometry (VITEK MS, bioMérieux, Marcy-l'Étoile, France) as *Actinomyces oris.* Antimicrobial therapy was switched to penicillin G 1.2 g 4 hourly. He was taken for surgery and had three vein grafts applied to the obtuse and marginal branches as well as a left internal mammary artery graft to the left anterior descending artery. In addition, an abscess around the RCA was de-roofed and a fistulous communication between the abscess cavity in the pericardium and the right atrium was closed. The initial RCA stent was hanging in the middle of the abscess cavity. Cultures of surgical tissues did not yield any growth. Unfortunately, no tissue material was submitted for histopathological examination. The post-operative course was unremarkable. The patient was discharged home with arrangements for outpatient daily intravenous ceftriaxone therapy for six weeks followed by oral amoxicillin to complete a total of six months of antimicrobial therapy. The patient remains well with no clinical, biochemical or radiological evidence of relapse of infection.

## Discussion

We herein report an unusual case of cardiac actinomycosis presenting several months after PCI. It is believed that coronary stent infection occur as a result of inoculation at the time of stent placement, or due to hematogenous spread from another source of infection [8]. Important risk factors for stent infection include older age, difficult vascular access, extended duration of the procedure and repeated catheterizations by the same vascular access site [6]. In this report, two PCI attempts, three months apart, were required to achieve successful recanalization. It is possible that excessive manipulation has contributed to the ensuing infective complication.

The final diagnosis of PCI-related cardiac actinomycosis is based on a combination of radiological, microbiological and intra-operative findings. Cultures of tissue obtained during surgery were negative. This is not surprising given that the patient received at least 4 days of intravenous piperacillin-tazobactam followed by 4 days of penicillin G prior to his surgery.

The clinical presentation of acute coronary syndrome and stent occlusion is common in patients with PCI-associated infections [[6], [7], [8]]. The presence of fever and high inflammatory markers, as reported here, should raise concern for infection as a rare but potentially serious complication of PCI [12]. The probable sequence of events in our patient is that a slowly growing actinomycotic abscess encasing the RCA eventually resulted in cardiac ischemia and clinical presentation with stable angina without evidence of infection. Subsequently, fistulization into the right atrium resulted in translocation of *Actinomyces* from the abscess cavity to the blood stream and was associated with systemic sepsis and the isolation of the bacteria from blood cultures.

We identified only one previous report of possible PCI-associated cardiac actinomycosis. The patient was a 75 year old man who had undergone PCI for coronary artery disease 4 months prior to his hospitalization, in addition to recent surgical intervention for bowel perforation. Echocardiogram showed evidence of a thickened pericardium and a large pericardial effusion. *A. israelii* was isolated from pericardial fluid cultures [15]. It is not clear this was related to the recent coronary intervention or from an intra-abdominal source.

Cardiac actinomycosis is generally rare. Our search of the literature yielded a total of 29 cases of cardiac actinomycosis (Table 1) [[15], [16], [17], [18], [19], [20], [21], [22], [23], [24], [25], [26], [27], [28], [29], [30], [31], [32], [33], [34], [35], [36], [37], [38], [39], [40], [41], [42], [43]]. The majority of cases were males and the median age was 45 years (range 14–81). The commonest site of involvement was the pericardium (15, 51.7 %) followed by one or more cardiac valves (12, 41.4 %). Right-sided valvular involvement was reported in two cases, both in association with intravenous drug use [35,43]. *A. israelii* (11, 37.9 %) and *A. odontolyticus* (4, 13.8 %) were the most frequently reported causative species, though speciation was not always available. Management involved surgical intervention in the majority (18, 62.1 %) of the reported cases. Moreover, beta-lactams were the most commonly used antimicrobial therapy agents, as single agents (14, 58.3 %) or in combination (8, 33.3 %). The main reason for use of beta-lactam alternatives was history of penicillin allergy [15,16] or in-vitro non-susceptibility of the isolated strains [17,41].

**Table 1:** Reports of cardiac actinomycosis.

Reference	Age (years)	Gender	Species	Sites involved	Notable medical history	Surgical Intervention	Antimicrobial therapy	outcome
Bellanti, 2017 [16]	45	Male	*A. israelii*	Pericardium	Recent percutaneous lung biopsy	Yes	Clindamycin	Alive
Broly, 2016 [17]	52	Female	*A. odontolyticus*	Pericardium	Asymptomatic dentigerous cyst	Yes	Doxycycline	Alive
Cole, 1982 [18]	24	Female	*Actinomyces sp.*	Pericardium	None	Yes	Penicillin	Alive
Fife, 1991 [19]	41	Female	*Actinomyces sp.*	Pericardium	None	Yes	Penicillin	Alive
Grundmann, 2010 [20]	66	Male	*A. neuii*	Aortic valve	Prosthetic aortic valve	No	Penicillin	Alive
Hamed, 1998 [21]	81	Male	*A. viscosus*	Aortic valve	None	No	Ceftizoxime	Alive
Huang, 1995 [22]	55	Female	*A. meyeri*	Mitral valve	None	No	Ampicillin-sulbactam	Alive
Jánoskuti, 2004 [23]	48	Female	*A. israelii*	Pericardium	None	Yes	Penicillin	Alive
Julian, 2005 [24]	43	Female	*A. viscosus*	Aortic valve	None	Yes	Ceftriaxone	Alive
Kottam, 2015 [25]	30	Female	*A. turicensis*	Eustachian valve and intra-abdominal	Intrauterine device insertion	Yes	Penicillin and imipenem	Alive
Lam, 1993 [26]	65	Male	*A. israelii*	Mitral valve	Rheumatic heart disease	No	Penicillin	Alive
Litwin, 1999 [27]	68	Male	*A. odontolyticus*	Pericardium and pleura	Gastrectomy for gastric carcinoma	yes	Ceftriaxone	Alive
Llenas-Garcia, 2012 [28]	20	Male	*A. israelii*	Pericardium and liver	Esophagectomy and colonic interposition	Yes	Imipenem and amikacin	Alive
Mack, 2014 [29]	61	Male	A. odontolyticus	Pericardium	Needle aspiration of Para tracheal lymph nodes	Yes	Piperacillin-tazobactam and ciprofloxacin	Died
Mac Neal, 1946 [30]	39	male	*A. septicus*	Mitral valve	None	No	Penicillin	Alive
Makaryus, 2005 [15]	75	Male	*A. israelii*	Pericardium	Percutaneous coronary intervention and colectomy	Yes	Doxycycline	Alive
Moffatt, 1996 [31]	48	Male	*A. meyeri*	Aortic valve	Rheumatoid arthritis	Yes	Penicillin	Alive
Mohan, 1974 [32]	51	Female	*A. israelii*	Pericardium	None	Yes	Not reported	Alive
Nishizawa, 2018 [33]	56	Male	A. meyeri	Pericardium and lung	Parkinson’s disease with psychosis	Yes	Penicillin and doxycycline	Alive
Oddo, 2007 [34]	34	Male	*Actinomyces sp.*	Mitral valve	Rheumatic heart disease	No	None	Died
Oh, 2005 [35]	33	Male	*A.* odontolyticus	Tricuspid valve	Intravenous drug use	No	Penicillin and metronidazole	Alive
Orloff, 1988 [36]	43	Male	*A. israelii*	Pericardium	Blunt chest trauma	Yes	Penicillin and clindamycin	Alive
Radu, 2018 [37]	14	Male	*A. israelii*	Lung and myocardium	None	No	None	Died
Sakaguchi, 2012 [38]	60	Male	A. druse	Pericardium and liver	None	Yes	Ampicillin-sulbactam	Alive
Shinagawa, 2002 [39]	42	Male	A. israelii	Pericardium and lung	None	Yes	Penicillin and minocycline	Alive
Slutzker, 1989 [40]	36	Male	*Actinomyces sp.*	Pericardium	None	Yes	Penicillin	Alive
Stokes, 1951 [41]	27	Female	*A. muris*	Mitral and aortic valves	Rheumatic heart disease	No	Chloramphenicol	Alive
Toom, 2018 [42]	55	Female	*A. israelii*	Mitral and aortic valve	Hypertrophic obstructive cardiomyopathy	No	Penicillin	Alive
Westling, 2002 [43]	40	Female	*A. funkei*	Tricuspid valve	Intravenous drug use	No	Cefuroxime, clindamycin and rifampin	Alive

Overall survival was remarkably good (26, 89.7 %). Two young patients were diagnosed from post-mortem cultures. One was a 17-year old man with rheumatic heart disease who died with mitral valve endocarditis, while the second patient was a 14 year old boy without any significant past medical history [34,37]. The third death was reported in a 61 year old patient with pericardial actinomycosis in association with metastatic squamous cell lung cancer [30]. In this report, early appropriate antimicrobial therapy, timely surgical intervention, removal of the infected tissue and stent and closure of the fistula all contributed to prompt clinical and microbiological response and overall successful outcome.

In summary, PCI-associated infection should be suspected in patients with ischemic manifestations associated with signs of systemic sepsis. Clinical evaluation should include blood cultures and cardiac imaging. Cardiac actinomycosis is rare. However, early recognition, appropriate antimicrobial therapy and surgical intervention are associated with excellent clinical outcomes.

## Funding

Qatar National Library.

## Consent

Written informed consent was obtained from the patient for publication of this case report and accompanying images. A copy of the written consent is available for review by the Editor-in-Chief of this journal on request.

## Author contribution

Writing the initial draft of the manuscript: Walaa Saeed, Mohamed Adam Conceptualization and supervision: Ali Omrani.

Medical management of the case: Walaa Saeed, Ali Omrani.

Revising the manuscript and literature review: Walaa Saeed, Mohamed Adam, Tasneem Abdallah, Ali Omrani.

The first authors (Walaa Saeed, Mohamed Adam) contributed equally to the writingand preparation of this article. Walaa Saeed, and Mohamed Adam have written the initial draft of the manuscript and performed the literature review. The draft was revised and updated by WS, MA with supervision from Tasneem Abdullah and Ali Omrani. WS and AO were part of the medical treating team. All the authors critically reviewed the initial and the final draft of the manuscript and approved it for submission.

## Role of the funding source

There is no role of Qatar National Library relevant to this case report.

## CRediT authorship contribution statement

**Walaa Saeed:** Writing - original draft, Data curation, Writing - review & editing, Visualization. **Mohammad Adam:** Writing - original draft, Data curation, Writing - review & editing, Visualization. **Tasneem A. Abdallah:** Data curation, Writing - review & editing. **Ali S. Omrani:** Conceptualization, Writing - review & editing, Supervision, Funding acquisition.

## Declaration of Competing Interest

The authors report no declarations of interest.

## References

[1] Könönen E., Wade W.G. (2015). Actinomyces and related organisms in human infections. Clin Microbiol Rev.

[2] Pulverer G., Schutt-Gerowitt H., Schaal K.P. (2003). Human cervicofacial actinomycoses: microbiological data for 1997 cases. Clin Infect Dis.

[3] Felz M.W., Smith M.R. (2003). Disseminated actinomycosis: multisystem mimicry in primary care. South Med J.

[4] Wong V.K., Turmezei T.D., Weston V.C. (2011). Actinomycosis. BMJ.

[5] Smego R.A., Foglia G. (1998). Actinomycosis. Clin Infect Dis.

[6] Kaufmann B.A., Kaiser C., Pfisterer M.E., Bonetti P.O. (2005). Coronary stent infection: a rare but severe complication of percutaneous coronary intervention. Swiss Med Wkly.

[7] Davidson L.J., Ricciardi M.J. (2018). Coronary artery perforation complicated by pericardial abscess formation: a clinical dilemma. Circ Cardiovasc Interv.

[8] Lai C.H., Lin Y.K., Lee W.L., Chang W.C. (2017). Coronary stent infection presented as recurrent stent thrombosis. Yonsei Med J.

[9] Sangolkar R., Ketana V.R.R., Sastry B.K.S. (2018). Coronary artery stent infection presenting as coronary cameral fistula: a case report. Eur Heart J Case Rep.

[10] Elieson M., Mixon T., Carpenter J. (2012). Coronary stent infections: a case report and literature review. Tex Heart Inst J.

[11] Bouchart F., Dubar A., Bessou J.P., Redonnet M., Berland J., Mouton-Schleifer D. (1997). *Pseudomonas aeruginosa* coronary stent infection. Ann Thorac Surg.

[12] Le M.Q., Narins C.R. (2007). Mycotic pseudoaneurysm of the left circumflex coronary artery: a fatal complication following drug-eluting stent implantation. Catheter Cardiovasc Interv.

[13] Gonda E., Edmundson A., Mann T. (2007). Late coronary stent infection: a unique complication after drug-eluting stent implantation. J Invasive Cardiol.

[14] Del Trigo M., Jimenez-Quevedo P., Fernandez-Golfin C., Vano E., Delgado-Bolton R., Alfonso F. (2012). Very late mycotic pseudoaneurysm associated with drug-eluting stent fracture. Circulation.

[15] Makaryus A.N., Latzman J., Yang R., Rosman D. (2005). A rare case of *Actinomyces israelii* presenting as pericarditis in a 75-year-old man. Cardiol Rev.

[16] Bellanti R., Chousou Pa, Pugh Pj. (2017). Pericardial actinomycosis in a patient with oesophageal dysmotility and autoantibodies. Br J Hosp Med (Lond).

[17] Broly E., Risse J., Maschino F., Wahl D. (2016). Cardiac tamponade due to *Actinomyces odontolyticus* originating from a dentigerous Cyst. J Oral Maxillofac Surg.

[18] Cole F.H., Jarrett C.L. (1982). Primary actinomycosis of the pericardium. South Med J.

[19] Fife T.D., Finegold S.M., Grennan T. (1991). Pericardial actinomycosis: case report and review. Rev Infect Dis.

[20] Grundmann S., Huebner J., Stuplich J., Koch A., Wu K., Geibel-Zehender A. (2010). Prosthetic valve endocarditis due to *Actinomyces neuii* successfully treated with antibiotic therapy. J Clin Microbiol.

[21] Hamed K.A. (1998). Successful treatment of primary Actinomyces viscosus endocarditis with third-generation cephalosporins. Clin Infect Dis.

[22] Huang K.L., Beutler S.M., Wang C. (1998). Endocarditis due to *Actinomyces meyeri*. Clin Infect Dis.

[23] Jánoskuti L., Lengyel M., Fenyvesi T. (2004). Cardiac actinomycosis in a patient presenting with acute cardiac tamponade and a mass mimicking pericardial tumour. Heart.

[24] Julian K.G., de Flesco L., Clarke L.E., Parent L.J. (2005). Actinomyces viscosus endocarditis requiring aortic valve replacement. J Infect.

[25] Kottam A., Kaur R., Bhandare D., Zmily H., Bheemreddy S., Brar H. (2015). Actinomycotic endocarditis of the eustachian valve: a rare case and a review of the literature. Tex Heart Inst J.

[26] Lam S., Samraj J., Rahman S., Hilton E. (1993). Primary actinomycotic endocarditis: case report and review. Clin Infect Dis.

[27] Litwin K.A., Jadbabaie F., Villanueva M. (1999). Case of pleuropericardial disease caused by *Actinomyces odontolyticus* that resulted in cardiac tamponade. Clin Infect Dis.

[28] Llenas-Garcia J., Lalueza-Blanco A., Fernandez-Ruiz M., Villar-Silva J., Ochoa M., Lozano F. (2012). Primary hepatic actinomycosis presenting as purulent pericarditis with cardiac tamponade. Infection.

[29] Mac Neal W.J., Blevins A., Duryee A.W. (1946). Clinical arrest of endocardial actinomycosis after 44 million units of penicillin. Am Heart J.

[30] Mack R., Slicker K., Ghamande S., Surani S.R. (2014). *Actinomyces odontolyticus*: rare etiology for purulent pericarditis. Case Rep Med.

[31] Moffatt S., Ahmen A.R., Forward K. (1996). First reported case of bacterial endocarditis attributable to *Actinomyces meyeri*. Can J Infect Dis.

[32] Mohan K., Dass S.I., Kemble E.E. (1974). Actinomycosis of pericardium. JAMA.

[33] Nishizawa S., Anan K., Tobino K., Okahisa M., Goto Y., Murakami K. (2018). Pulmonary actinomycosis attributable to *Actinomyces meyeri* presenting as cardiac tamponade: a case report. Multidiscip Respir Med.

[34] Oddo B.D., Ayala R.F. (2007). Actinomycotic infective endocarditis of the mitral valve. Anatomoclinical case and review of literature. Rev Chilena Infectol.

[35] Oh S., Havlen P.R., Hussain N. (2005). A case of polymicrobial endocarditis caused by anaerobic organisms in an injection drug user. J Gen Intern Med.

[36] Orloff J.J., Fine M.J., Rihs J.D. (1988). Acute cardiac tamponade due to cardiac actinomycosis. Chest.

[37] Radu C.C., Camarasan A., Podila C.M., Perju-Dumbrava D. (2018). Sudden death of a teenager caused by *Actinomyces israelii*: a case report. Iran J Public Health.

[38] Sakaguchi Y., Isowa N., Nakazaki H., Takeda K., Tokuyasu H., Saitoh Y. (2012). Acute cardiac tamponade caused by the extension of multiple hepatic actinomycotic abscesses. Intern Med.

[39] Shinagawa N., Yamaguchi E., Takahashi T., Nishimura M. (2002). Pulmonary actinomycosis followed by pericarditis and intractable pleuritis. Intern Med.

[40] Slutzker A.D., Claypool W.D. (1989). Pericardial actinomycosis with cardiac tamponade from a contiguous thoracic lesion. Thorax.

[41] Stokes J.F., Gray I.R., Stokes E.J. (1951). *Actinomyces muris* endocarditis treated. With chloramphenicol. Br Heart J.

[42] Toom S., Xu Y. (2018). Hemolytic anemia due to native valve subacute endocarditis with *Actinomyces israelii* infection. Clin Case Rep.

[43] Westling K., Lidman C., Thalme A. (2002). Tricuspid valve endocarditis caused by a new species of actinomyces: *Actinomyces funkei*. Scand J Infect Dis.

